# Association Between Physical Activity and Mortality in Men with or at Risk of Prostate Cancer: A Systematic Review

**DOI:** 10.3390/healthcare14131998

**Published:** 2026-07-05

**Authors:** Nacho García-Miralles, Irene Martínez-García, Irene Marcilla-Toribio, Andrea Herreros-Solano, Jaime Fernández-Bravo-Rodrigo, Silvana Patiño-Cardona, Elena Moreno-Charco, Amparo María Ortega-Armiñana, María Gregori-Navarro, Carlos Pascual-Morena

**Affiliations:** 1 Health and Social Research Center, University of Castilla-La Mancha, 16071 Cuenca, Spain; nacho.garcia@alu.uclm.es (N.G.-M.); irene.marcilla@uclm.es (I.M.-T.); andrea.herreros@alu.uclm.es (A.H.-S.); silvana.patino@alu.uclm.es (S.P.-C.); elena.moreno9@alu.uclm.es (E.M.-C.); amparomaria.ortega1@alu.uclm.es (A.M.O.-A.); maria.gregori1@alu.uclm.es (M.G.-N.); carlos.pascual@uclm.es (C.P.-M.); 2 Faculty of Nursing, University of Castilla-La Mancha, 02006 Albacete, Spain; 3 Health, Gender, and Social Determinants Research Group, Health and Social Research Center, University of Castilla-La Mancha, 16071 Cuenca, Spain; 4 CarVasCare Research Group, Faculty of Nursing, University of Castilla-La Mancha, 16001 Cuenca, Spain; jaime.fernandezbravo@alu.uclm.es; 5 Pharmacy Service, Hospital Virgen del Castillo, 30510 Yecla, Spain; 6 Cuenca Primary Care Management, Hospital Universitario de Cuenca, Castilla-La Mancha Health Service, 16003 Cuenca, Spain; 7 Provincial Coordination and Inspection Service, SESCAM, 13001 Ciudad Real, Spain; 8 Pharmacy Department, Hospital General Universitario de Castellón, 12004 Castellón, Spain; 9 Pharmacy Department, Sagunto Hospital, 46520 Valencia, Spain

**Keywords:** healthy lifestyle, exercise, prognosis, mortality, cancer, review

## Abstract

Introduction: Prostate cancer (PC) is a highly prevalent malignant tumour associated with significant morbidity and mortality. While physical activity has been linked to a lower risk of PC and exercise has been shown to reduce mortality, the evidence for the association between physical activity and mortality is limited. Objective: This study aimed to assess the association between physical activity and mortality risk in men with or at risk of PC. Methods: A systematic search was conducted in Medline, Scopus and Web of Science from inception until April 2026. Observational studies analysing physical activity and all-cause and PC-specific mortality were included. The data were synthesised and interpreted using a synthesis without meta-analysis (SWiM) approach. The quality of the studies was assessed using the NHLBI tool. The certainty of the evidence was assessed using the GRADE framework. Results: Fifteen observational studies were included. The hazard ratio (HR) was the predominant effect measure. Physical activity was associated with a reduction in all-cause mortality (HRs: 0.40–0.88; highest versus lowest categories), and a dose–response gradient was observed within two cohorts. Associations with PC-specific mortality were less consistent, with significant inverse findings concentrated in post-diagnosis assessments. The quality of the studies was generally poor, and the certainty of the evidence was very low for both outcomes. Conclusions: Physical activity was associated with lower all-cause mortality risk in men with or at risk of PC, and the most consistent inverse estimates were observed in post-diagnostic assessments. These findings are observational and should not be interpreted as a clinical recommendation. A dose–response pattern was noted within individual studies, although the certainty of evidence was very low for this outcome. Additionally, evidence for PC-specific mortality was inconsistent and of very low certainty. Prospective studies with standardised, objective measures of physical activity are required.

## 1. Introduction

Prostate cancer (PC) is a malignant tumour of epithelial origin that develops in the prostate gland. It is characterised by abnormal cellular proliferation and usually progresses slowly in the early stages, although it has the capacity to become invasive and metastatic [1]. Histologically, PC is predominantly an acinar adenocarcinoma; less frequent subtypes, such as ductal adenocarcinoma and tumours with neuroendocrine differentiation, are associated with more aggressive biological behaviour and poorer prognoses [2,3,4]. Around 1.4 million new cases are diagnosed worldwide each year, primarily affecting men over 65. Consequently, PC is considered the second most common cancer, affecting over 10 million men worldwide. This makes PC the fifth leading cause of cancer death globally, with 396,000 deaths per year [5,6,7,8,9,10]. Furthermore, PC and its treatments can result in long-term urinary incontinence and erectile dysfunction, particularly following prostatectomy or radiotherapy. Metastatic spread to the bones and other organs, on the other hand, is associated with severe bone pain, pathological fractures, and a reduction in life expectancy and quality of life [11,12,13,14].

PC has a multifactorial aetiology, and its pathogenesis results from the interaction between advanced age, androgenic hormonal stimulation, and genetic susceptibility. Lifestyle factors such as excessive alcohol consumption, smoking, a Western-style diet, obesity, chronic prostatic inflammation and the presence of premalignant lesions contribute to the development of invasive carcinoma [15,16,17,18,19]. Specifically, obesity, particularly central adiposity, has been consistently associated with an increased risk of advanced and aggressive PC. Physical activity has a protective effect, improving hormonal, inflammatory and metabolic profiles. As physical activity is inversely associated with obesity, it may reduce PC-specific mortality through interconnected biological mechanisms. Firstly, physical activity reduces body fat and improves insulin sensitivity, thereby minimising chronic exposure to insulin and insulin-like growth factor 1 (IGF-1), which are metabolic mediators involved in tumour proliferation and PC progression. Physical activity also influences the metabolic and hormonal environment, reducing inflammation and improving immune function, thereby modulating key processes in tumour progression. Furthermore, physical activity induces epigenetic changes that regulate genes involved in cell-cycle control and apoptosis [20,21,22,23,24].

At the molecular level, PC is critically dependent on androgen stimulation and the transcriptional activation of the androgen receptor (AR). It has been hypothesised that exercise and physical activity can modulate the gonadal endocrine environment by reducing androgen signalling and AR activity in the prostatic parenchyma. However, evidence from human tissue is limited. Furthermore, physical activity exerts homeostatic control over the insulin-like growth factor 1 (IGF-1) axis, reducing circulating IGF-1 concentrations and increasing IGF-binding protein 3 (IGFBP-3) levels in cancer patients. This has also been associated with a lower risk of PC. The increase in IGFBP-3 limits the bioactive fraction of free IGF-1, consequently reducing the activation of intracellular mitogenic cascades dependent on IGF-1. These cascades include the mitogen-activated protein kinase (MAPK/ERK) and phosphatidylinositol 3-kinase (PI3K/Akt/mTOR) pathways, which are considered to be determinants of prostate tumour progression [25,26,27].

Previous systematic reviews and meta-analyses have examined the relationship between physical activity and the risk of developing PC. These studies have indicated a potential positive association, particularly in relation to advanced stages of the disease [20]. Furthermore, a recent meta-analysis assessed the effect of physical exercise on survival in patients with PC [28]. Finally, a systematic review from 2018 based on four studies showed a reduction in mortality [29]. Since then, several studies have been published, enabling an updated systematic review and improving the evidence on this topic. Thus, the main contribution of this systematic review is a revised, stratified, non-pooled synthesis that distinguishes pre-diagnostic from post-diagnostic exposure windows rather than addressing a fundamentally different research question. Therefore, this systematic review aimed to assess the association between physical activity and mortality risk in men with or at risk of PC.

## 2. Materials and Methods

This study was conducted in accordance with the Cochrane Handbook for Systematic Reviews of Interventions and the Preferred Reporting Items for Systematic Reviews and Meta-Analyses (PRISMA) [30,31]. The study protocol was registered in the PROSPERO database (CRD420261380464).

### 2.1. Search Strategy

A systematic search was conducted in Medline (via PubMed), Scopus and Web of Science from inception to April 2026. The search strategy was designed to identify studies evaluating the association between physical activity and the risk of PC mortality. Search terms relating to PC, physical activity and mortality were used, applying the PECO (participants, exposure, comparator and outcomes) structure and Boolean operators (AND and OR). No filters were applied for date, language, or study design. An open, non-systematic search was also performed in Google Scholar, and grey literature was examined, including the Networked Digital Library of Theses and Dissertations and Open Grey. Given the difficulty of systematically documenting open searches, sources such as these are reported narratively rather than as reproducible search strings. These searches were carried out in April 2026. The reference lists of the included studies and relevant reviews were manually screened to identify any further eligible articles. The search strategy is described in Section S1.

The systematic search was performed independently by two authors (NG-M and CP-M) and any disagreements were resolved by a third author (IM-G).

### 2.2. Inclusion and Exclusion Criteria

Observational studies that assessed the association between physical activity and mortality in men with or at risk of PC were included.

The inclusion criteria were as follows: (1) Population: adult men, encompassing both general population cohorts, which are used to assess pre-diagnostic physical activity and PC-specific mortality, and clinical cohorts of cancer survivors (i.e., patients with a confirmed pathological diagnosis of PC); (2) Exposure: physical activity, ascertained via questionnaires or equivalent measures (total, recreational or occupational activity, or activity of moderate or vigorous intensity); (3) Comparator: comparison of different physical activity levels (high vs. low), or of categories defined within each study; (4) Outcome: all-cause or PC-specific mortality in men with or at risk of PC; (5) Design: observational studies. No language restrictions were applied.

Exclusion criteria were: (1) posters, conference abstracts, letters to the editor, editorials or comments reporting partial results, and (2) cross-sectional studies, case series or case studies that did not provide appropriate risk estimates, such as hazard ratios (HRs), risk ratios (RRs) or odds ratios (ORs).

Study selection was performed by two authors (NG-M and CP-M), and disagreements were resolved by consensus or consultation with a third author (IM-G).

### 2.3. Data Extraction

The following information was extracted from the observational studies included in the systematic review: (1) author and year of publication; (2) country or region of the study; (3) study design; (4) sample size, including by PC stage; (5) mean age of participants; (6) method used to assess physical activity; (7) assessment timing (pre- or post-diagnostic) and cohort type (general population or survivors); (8) follow-up duration; (9) comparison type (categorical or linear analysis); and (10) outcomes (risk of all-cause and PC-specific mortality).

Data extraction was performed by two authors (NG-M and CP-M), and disagreements were resolved by consensus or by a third author (IM-G).

### 2.4. Assessment of Study Quality

The quality of the studies included in the systematic review was assessed using the National Heart, Lung, and Blood Institute (NHLBI) Study Quality Assessment Tool [32]. This tool evaluates various methodological and statistical aspects related to population selection, exposure and outcome measurement, and control of confounding factors. Studies were classified as ‘good’ if fewer than two items were at high risk, ‘fair’ if two items were at high risk, and ‘poor’ if more than two items were at high risk.

Quality assessment was performed by two authors (NG-M and CP-M), and disagreements were resolved by consensus or by a third author (IM-G).

### 2.5. Assessment of Certainty of Evidence

The GRADE (Grading of Recommendations, Assessment, Development and Evaluation) system was used to assess the certainty of the evidence [33,34]. This tool classifies evidence as being of either high, moderate, low or very low certainty. It considers factors such as the design of the included studies, the risk of bias, the consistency and precision of the results, the indirectness of the evidence, the effect size and the possible presence of uncontrolled confounding or publication bias.

### 2.6. Data Synthesis

A specific ad hoc table was developed to summarise the observed associations in the included studies. The estimates were expressed as risk measures (HR, RR or OR), along with their corresponding 95% confidence intervals (CI).

A synthesis without meta-analysis (SWiM) was then conducted in accordance with the SWiM reporting guidelines to evaluate the included studies [35]. Vote counting based on the direction of effect was used as the synthesis method. For each cohort, the extracted effect estimate was classified as an inverse association (where higher physical activity is associated with a lower mortality risk), a positive association (where higher physical activity is associated with a higher mortality risk) or not statistically significant. To minimise residual confounding, adjusted effect estimates were prioritised over unadjusted models.

To ensure the reproducibility of the results, a single estimate was pre-specified for each study, outcome and timing stratum. This was selected according to the following hierarchy: (1) the adjusted categorical contrast comparing the highest versus the lowest exposure category, unless the authors used a different reference comparison which made it impossible to estimate the association against the group with less physical activity; (2) where multiple physical activity domains were reported, the total or overall physical activity measure; (3) where both overall and subgroup estimates were available, the overall estimate; and (4) where both categorical and continuous estimates were reported, the categorical contrast. Direction was determined from the 95% CI: inverse (upper bound <1), positive (lower bound >1) or non-significant (CI crossing 1). An effect-direction harvest plot was constructed to display the direction of each selected estimate alongside sample size (bar height = log_10_ N), stratified by outcome and timing of exposure assessment. Additionally, an albatross plot was generated to depict the relationship between sample size and statistical significance for each estimate, with reference curves indicating approximate effect-size contours.

To address clinical heterogeneity, the synthesis was stratified by the timing of the physical activity assessment, with a distinction made between pre-diagnostic and post-diagnostic exposure windows. Within each stratum, the studies were also classified by cohort type (general population versus PC-specific/survivor cohorts) to enable the direction of effect to be enumerated separately by population source. Potential dose–response gradients were also assessed by examining cohorts that reported continuous physical activity increments or multiple ordinal exposure categories. The results were considered statistically significant when *p* < 0.05.

### 2.7. Modifications to the Registered Protocol CRD420261380464

Two modifications were made to the original PROSPERO registration. First, the search strategy was expanded to include grey literature sources, which were not specified in the protocol. Second, the planned quantitative synthesis was revised. Although a random-effects meta-analysis had initially been intended, a SWiM approach was ultimately adopted following data extraction, upon confirmation that the conditions described below precluded meaningful pooling. This decision was taken for three reasons: (i) physical activity was quantified using non-standardised metrics across cohorts, which precluded the construction of a common effect estimate without making arbitrary unit conversions; (ii) including multiple comparisons from the same study that shared a common reference group introduced a unit-of-analysis error that inflated precision. Selecting a single estimate per study reduced the number of poolable estimates per stratum below the threshold required for stable random-effects modelling, and (iii) there was substantial clinical and methodological heterogeneity across cohorts. This included differences in population type, outcome ascertainment and follow-up duration. This further precluded meaningful pooling. Consequently, the planned statistical analyses, including the Hartung–Knapp–Sidik–Jonkman and DerSimonian–Laird models, meta-regression and assessment of publication bias, were not performed. To display the direction, magnitude, and precision of the selected estimates without pooling, an effect-direction harvest plot and an albatross plot were added to the synthesis.

## 3. Results

**Figure 1:**
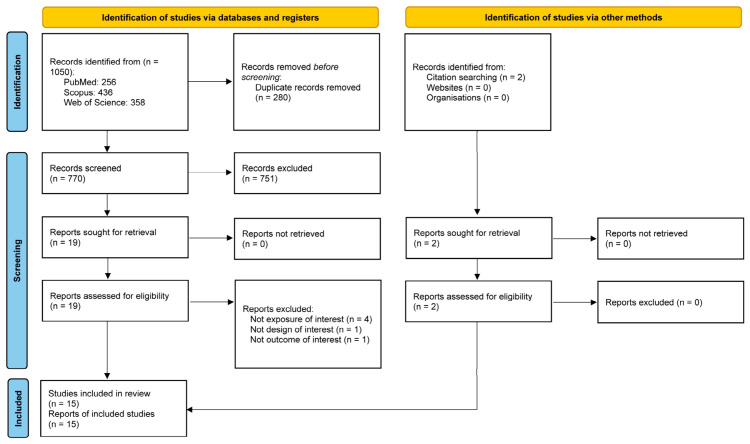
PRISMA flowchart of study selection.

A total of 1050 records were retrieved, of which 280 were duplicates. A total of 770 articles were screened by title and abstract, and 21 of these were assessed in full (19 from PubMed, Scopus and Web of Science, and 2 from open search or grey literature). Fifteen studies were ultimately included in the systematic review (Figure 1, Table 1) [36,37,38,39,40,41,42,43,44,45,46,47,48,49,50], while six were excluded for justified reasons (Table S1) [51,52,53,54,55,56].

Most of the studies were conducted in the United States (n = 7), followed by Sweden (n = 3). Individual studies were also conducted in Canada, Norway, the United Kingdom, Puerto Rico and Italy. In terms of study design, prospective cohort studies predominated (n = 11), while the remaining studies (n = 4) adopted a retrospective approach. Sample sizes varied considerably, ranging from 777 to 404,249 participants. Participants’ mean age predominantly fell within the range of 60 to 72 years, although younger populations from 41 years of age were also included.

In terms of assessing physical activity, most studies used validated self-reporting questionnaires such as the Health Professionals Follow-up Study (HPFS) or the Physical Activity Lifetime Questionnaire (LTPAQ). These questionnaires quantified exposure using metabolic equivalents of task (MET-h/week) or intensity levels. One study included objective measurements through triaxial accelerometry [49]. Most studies differentiated physical activity by pre- and/or post-diagnosis periods, evaluating its association with all-cause and PC-specific mortality. The characteristics of the included studies are described in Table 1 and Table S2.

### 3.1. Assessment of Study Quality

According to the NHLBI quality assessment tool for observational studies, the overall quality was predominantly rated as poor. Overall, 1 out of 15 studies (6.7%) was rated as good, 3 out of 15 studies (20%) as fair, and 11 out of 15 studies (73.3%) as poor. The main limitations in the latter studies were a lack of repeated physical activity assessments and an absence of statistical justification for sample size. The item-level analysis revealed 100% compliance with the definition of objectives and selection criteria. However, areas for improvement were identified in the justification of statistical power (item 5) and the participation rate (item 3). These were either not reported or reported insufficiently in a large proportion of studies. Despite these limitations, multivariable adjustment for confounding variables was performed in all studies. The results of the quality assessment are presented in Table S3.

### 3.2. Assessment of Quality of Evidence

The certainty of the evidence for the analysed outcomes was assessed using the GRADE framework. The certainty of the evidence was rated as very low for both all-cause and PC-specific mortality. This was due to the observational nature of the studies and the serious risk of bias. For all-cause mortality, further downgrading was applied due to imprecision, as the absence of a pooled effect estimate meant that the precision of the overall association could not be formally evaluated, and several individual study CIs were wide. A dose–response gradient was identified for all-cause mortality within two cohorts reporting multiple ordinal exposure categories [43,49]. Both cohorts showed a consistent inverse gradient, which warranted a +1 upgrade under the GRADE criteria for observational evidence. However, two domains (risk of bias and imprecision) each applied a −1 downgrade, resulting in a net certainty of evidence rating of ‘very low’. Inconsistency was rated as serious for PC-specific mortality, given the heterogeneous direction and magnitude of estimates across cohorts, with non-significant findings predominating in pre-diagnostic strata. The assessment of evidence quality is presented in Table S4.

### 3.3. Findings of Systematic Review

**Figure 2:**
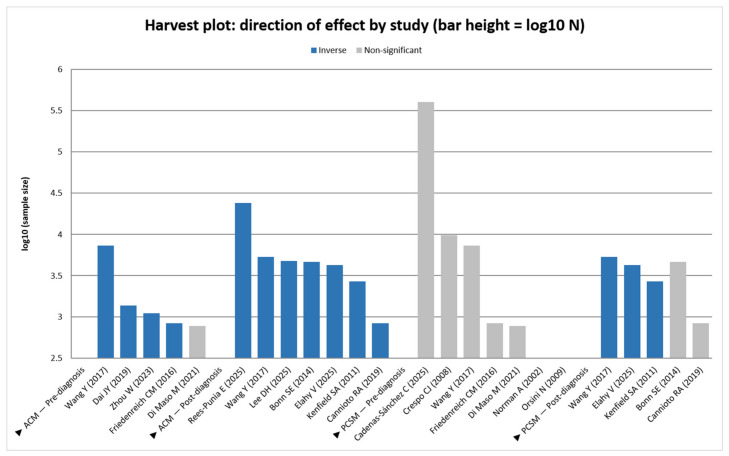
Effect-direction harvest plot of studies examining physical activity and mortality in men with, or at risk of, prostate cancer [36,37,38,39,40,41,42,43,44,45,46,47,48,49,50].

Table 2 summarises the results of the included studies assessing the association between physical activity and mortality in PC. In addition, Figure 2 displays the harvest plot of selected estimates by stratum. Figure S1 presents an albatross plot relating the sample size and statistical significance for each estimate, with reference curves indicating approximate effect-size contours. For all-cause mortality, the direction of effect was inverse in four of the five pre-diagnostic strata (n = 11,394) and in all seven post-diagnostic strata (n = 46,496). For PC-specific mortality, no pre-diagnostic stratum showed an inverse direction (0 of 7); three of five post-diagnostic strata did (n = 17,712). No stratum in any analysis showed a positive (harmful) direction of effect. One study contributed to both temporal strata, as it reported pre- and post-diagnostic estimates separately [48].

When the synthesis was further stratified by cohort type within each timing window, all five pre-diagnostic strata for all-cause mortality and all seven post-diagnostic strata for both outcomes were derived from survivor cohorts; pre-diagnostic strata for PC-specific mortality comprised four general-population cohorts [37,39,45,46] and three survivor cohorts [40,42,48], with no inverse direction observed in either subgroup (0 of 4 and 0 of 3, respectively). This further stratification, together with cohort-type tagging in Table 2 and in the harvest plot (Figure 2), confirms that the lack of inverse association for pre-diagnostic PC-specific mortality is consistent across cohort types and does not reflect a particular feature of general-population cohorts alone.

Fifteen studies assessing the association between physical activity and mortality in PC were identified. HR was the predominant effect measure, used in 11 studies; one study reported OR [39], two reported RR [45,46], and one reported a subdistribution hazard ratio (sHR) in the presence of competing risks [37]. Nearly all estimates corresponded to categorical comparisons (MET-hours/week categories, quartiles, time thresholds, or active versus inactive schemes); only one study provided a linear trend estimate per one-point increment on the physical activity component scale (HR for all-cause mortality = 0.90; 95% CI: 0.86–0.94; HR for PC-specific mortality = 0.82; 95% CI: 0.73–0.92) [41].

The narrative description below covers all comparisons reported by each study, whereas the formal vote (Figure 2) used the single pre-specified estimate per stratum described in Section 2.6. Regarding all-cause mortality, the 11 studies assessing this outcome predominantly showed an inverse association, with inverse HRs ranging from 0.40 (≥48 MET-hours/week versus <3) to 0.88 [43,44,48]. The only globally non-significant comparison contrasted high versus low/moderate activity (HR = 0.79; 95% CI: 0.59–1.07) [40]. Within studies showing an overall inverse association, the lowest exposure categories did not always reach significance (e.g., 3–<9 MET-hours/week: HR = 0.88; 95% CI: 0.74–1.06 [43]; 1.0–1.5 h/day: HR = 0.90; 95% CI: 0.69–1.16 [49]).

For PC-specific mortality, the studies yielded more heterogeneous results, with a predominance of non-significant estimates. Significant inverse associations were concentrated in post-diagnosis assessments: walking ≥20 min/day (HR = 0.61; 95% CI: 0.43–0.87) and exercise ≥1 h/week (HR = 0.68; 95% CI: 0.48–0.94) [36]; a linear trend estimate (HR = 0.67; 95% CI: 0.54–0.84) [41]; ≥48 MET-hours/week (HR = 0.46; 95% CI: 0.23–0.92) and ≥3 h/week of vigorous activity (HR = 0.39; 95% CI: 0.18–0.84) [44]; and post-diagnosis physical activity (HR = 0.69; 95% CI: 0.49–0.95) [48]. Estimates derived from occupational activity (sHR = 0.92–0.96) [37], quartile-based comparisons using ORs (Q2–Q4: 0.99–1.34) [39], and RRs (0.72–0.99) [45,46] did not reach statistical significance.

Assessment by activity level showed a dose–response gradient for all-cause mortality in studies with multiple categories: HRs ranged from 0.88 (3–<9 MET-hours/week) to 0.61 (≥48 MET-hours/week) [43], and from 0.90 (1.0–1.5 h/day) to 0.57 (≥2.0 h/day) [49], with the largest magnitudes observed in the highest exposure categories (e.g., ≥48 MET-hours/week: HR = 0.40 for all-cause mortality [44]). For PC-specific mortality, this gradient was inconsistent: quartile-based comparisons showed neither monotonicity nor significance (OR ranging from 0.99 to 1.34 [39]; RR ranging from 0.72 to 0.98 [46]).

With respect to the timing of measurements, inverse associations were more consistent and more frequently significant when physical activity was assessed post-diagnosis, for both all-cause mortality [36,41,43,44,47,48] and PC-specific mortality [36,41,44,48]. In pre-diagnosis assessments, inverse associations for all-cause mortality persisted in several studies [42,48,49,50], whereas for PC-specific mortality most estimates were non-significant [37,39,40,42,45,46]. The only study providing both temporal windows for the same outcome reported a non-significant pre-diagnosis HR for PC-specific mortality (0.95; 95% CI: 0.75–1.21) versus a significant post-diagnosis HR (0.69; 95% CI: 0.49–0.95), with pre-diagnosis significance observed only in the low-risk tumour subgroup (HR = 0.63; 95% CI: 0.43–0.91) [48].

Regarding the type of physical activity assessed, the estimates derived from occupational activity [37] yielded no significant associations with PC-specific mortality (sHR = 0.92–0.96), whereas recreational or leisure-time physical activity, particularly when quantified in MET-hours/week [41,43,44,47,48], showed more consistent inverse associations with both outcomes. Similarly, specific modalities such as walking [36,44] and structured exercise [36] were associated with inverse estimates for all-cause and, in some comparisons, PC-specific mortality. Conversely, broader or less specific categorisations of activity level (e.g., high versus low/moderate [40], high versus sedentary [45]) tended to yield non-significant estimates. No clear differential pattern emerged for vigorous activity as a separate domain: one study reported a significant inverse association with all-cause mortality [50], whilst another found no association with PC-specific mortality [39].

## 4. Discussion

Our findings show that the direction of effect was predominantly inverse for all-cause mortality across studies, with a dose–response pattern observed within two cohorts. The evidence for PC-specific mortality was less consistent. The certainty of the evidence was rated as very low for both outcomes under GRADE, which precludes us from drawing firm conclusions about the magnitude of any association or the differential effect of vigorous-intensity physical activity.

When interpreting these findings, it is important to consider that pre-diagnostic and post-diagnostic physical activity do not represent a single, interchangeable exposure. Rather, they capture distinct biological, behavioural and clinical processes. Pre-diagnostic activity, which is usually evaluated in general population cohorts, represents aetiological exposure. Its association with mortality is mediated by long-term effects on tumour initiation and the likelihood of developing aggressive or lethal disease. Consequently, it is susceptible to reverse causation, as subclinical disease may reduce activity prior to diagnosis. Post-diagnostic activity, as observed in survivor cohorts, is a prognostic factor: it affects an established tumour and host factors that influence progression, treatment tolerance and competing causes of death. These exposures differ behaviourally, since a diagnosis often prompts changes in activity, and clinically, since post-diagnostic activity depends on disease stage, treatment and functional status. Therefore, the consistent inverse associations observed for post-diagnostic activity should not be interpreted as evidence that activity is ‘more beneficial’ after diagnosis, but rather as an indication of distinct underlying mechanisms and differing susceptibility to bias in the two time periods. Pooling them into a generalised ‘physical activity’ effect would obscure these differences, which is why the synthesis was stratified by timing. This distinction is reflected in the results. Whereas inverse associations for all-cause mortality were observed in both pre- and post-diagnostic survivor cohorts, the absence of an inverse association for PC-specific mortality in the pre-diagnostic strata was consistent in both the general population and survivor cohorts. This suggests that the null finding is not due to cohort type alone.

According to our findings, the inverse estimates for all-cause mortality across studies clustered between HRs of approximately 0.50 and 0.80, with isolated estimates as low as 0.40 in the highest exposure categories. This range is consistent with that reported in previous meta-analyses [28,29,57] and aligns with broader epidemiological evidence supporting the overall role of physical activity in reducing cancer mortality [58]. As some authors have proposed [59], the considerable heterogeneity identified in our study is an expected phenomenon, mainly due to variability in measurement methods and differences in the baseline profiles of the studied populations. With regard to the extreme values observed, one study reported a greater-than-average risk reduction of 61%. This finding may be explained by the high level of vigorous physical activity assessed, which is consistent with the dose–response effect of physical activity described elsewhere [44,60,61]. This pattern is consistent with data suggesting that higher levels of physical activity after diagnosis are associated with improved survival [36,47]. Finally, the consistency of this benefit in long-term cohorts, such as those with prolonged follow-up [42,48], reinforces the proposal by one study that physical activity and exercise could play a therapeutic role in patients with PC [62].

Furthermore, for PC-specific mortality, the direction of effect was less consistent across studies, with inverse estimates concentrated in post-diagnostic assessments of survivor cohorts and largely null estimates in pre-diagnostic assessments of general-population cohorts. This pattern is consistent with the available evidence, with vigorous activity (≥3 h per week) being associated with a 61% reduction in disease-specific mortality, highlighting the importance of intensity [44]. Several studies have confirmed an inverse association between post-diagnostic physical activity and disease-specific mortality, even at moderate intensity levels [36,48], and these findings have been replicated in large-scale studies with prolonged follow-up [42,47].

In addition, a dose–response gradient has been observed, with a progressive reduction in risk as the volume of physical activity increases [43]. There is also evidence of lower progression to metastatic-lethal disease among more active individuals [50]. More broadly, both earlier and more recent meta-analyses have confirmed similar associations between physical activity and reduced cancer mortality [28,57], while studies specifically focused on PC provide additional evidence across different clinical settings and further support the role of intensity in tumour progression [63]. Finally, clinical reviews have established exercise as a relevant intervention in oncological management [62].

At the physiological level, there are multiple interrelated mechanisms that may explain the association between physical activity and lower PC mortality. Firstly, physical activity reduces chronic systemic inflammation, and together with improved insulin sensitivity and decreased levels of IGF-1, this contributes to limiting cellular proliferation and promoting tumour apoptosis. These metabolic and hormonal effects are integrated with the modulation of the endocrine environment, which is particularly relevant in hormone-sensitive tumours such as PC [64,65,66,67].

In addition, physical activity boosts the immune system by increasing the activity of natural killer cells and cytotoxic T lymphocytes. This strengthens immune surveillance against tumour cells [68,69]. Concurrently, reductions in adiposity and improvements in the metabolic profile decrease the secretion of pro-inflammatory adipokines, thereby further reducing systemic inflammation [70,71]. In this context, exercise-induced antioxidant adaptations limit cellular damage and mutagenesis. Meanwhile, the release of myokines acts as a mechanism of inter-organ communication with potential anti-tumour effects [72,73]. These systemic processes are accompanied by modifications to the tumour microenvironment, including changes to angiogenesis and cellular signalling, as well as interactions with the stroma [74,75]. Improved tissue perfusion and oxygenation also reduce tumour hypoxia and biological aggressiveness [76,77,78].

Specifically in the context of PC, systematic exercise modulates the androgenic endocrine axis by inducing a decrease in circulating levels of free testosterone and dihydrotestosterone, which reduces the transcriptional activation of the androgen receptor in malignant prostate cells. Furthermore, exercise-induced elevation of IGF-binding protein 3 (IGFBP-3) selectively sequesters free IGF-1, thereby blocking the mitogenic signalling cascades of the PI3K/Akt/mTOR pathway. In terms of the muscular secretome, certain myokines, such as oncostatin M (OSM) and secreted protein acidic and rich in cysteine (SPARC), have shown anti-tumour properties in prostate cell lines (e.g., LNCaP). These myokines promote cell-cycle arrest at the G1 phase via activation of the p38 MAPK pathway and suppress cell migration by inhibiting Akt phosphorylation [26,27,79,80,81].

The findings of the present study have important implications for clinical practice and research. From a clinical perspective, the available evidence supports considering physical activity as a non-pharmacological intervention in the management of patients with PC. Although inverse associations were more consistent in post-diagnostic assessments of survivor cohorts than in pre-diagnostic assessments of general-population cohorts, this should be interpreted with caution, as the two strata derive from non-comparable populations and exposure frameworks. It is possible that individuals who initiate physical activity after diagnosis have a poorer initial condition and therefore a greater capacity for improvement, whereas those who are already physically active may start from a better baseline status with less potential for further prognostic gain.

In this regard, integrating structured exercise programmes into routine clinical practice aligns with recommendations from major oncology guidelines, which advocate exercise prescription as part of a comprehensive therapeutic approach [82,83]. However, the variability in physical activity protocols reported in the literature highlights the need to personalise recommendations. This should take into account factors such as the intensity, duration and timing of the intervention, as well as the clinical characteristics of the patients [84,85]. Furthermore, improving exposure assessment through the use of objective tools, such as accelerometry, is a priority to reduce the measurement bias associated with self-reported questionnaires [86].

From a research perspective, these findings emphasise the importance of randomised controlled trials in establishing causal relationships and defining the optimal dose of physical activity. Future trial designs should prioritise decentralised or hybrid pragmatic frameworks in order to address the challenges inherent in this clinical population. These challenges include age-related recruitment barriers, the need for long-term adherence to exercise protocols during concurrent oncological therapies, and the prolonged tracking required for robust survival outcome ascertainment.

### Limitations

Several limitations should be noted. The considerable variability in the reported associations for all-cause mortality, together with the inclusion of general population and cancer survivor cohorts within the same grouping, is consistent with the very low GRADE certainty rating assigned to this outcome. Reliance on self-reported physical activity questionnaires introduces recall and classification bias. This is compounded in the pre-diagnostic subgroup by the conflation of prospective and retrospective assessments. Furthermore, the lack of standardised exercise quantification complicates cross-study comparison. Residual confounding factors, such as healthy-user bias and immortal time bias in post-diagnostic assessments, cannot be ruled out. Finally, the search was restricted to Medline, Scopus and Web of Science, excluding Embase. Although grey literature sources were consulted, the inherent difficulty of systematically documenting open searches limits the reproducibility of this aspect. The predominance of Caucasian cohorts from North America and Scandinavia further limits the generalisability of the results.

## 5. Conclusions

The findings of this systematic review suggest that physical activity is associated with a predominantly inverse direction of effect on all-cause mortality in men with or at risk of PC, with the most consistent inverse estimates observed in post-diagnostic assessments of survivor cohorts. The association with PC-specific mortality was less consistent. Moreover, a dose–response pattern was observed for all-cause mortality within two individual cohorts, with greater reductions associated with higher exposure categories. However, this finding should be interpreted with caution, given the limited number of studies reporting multiple ordinal categories. Furthermore, the certainty of the evidence was very low for both outcomes according to the GRADE approach, and the strictly observational design of the available evidence precludes firm causal inferences. Therefore, these findings should not be translated into clinical recommendations. Interpretation must remain stratified because pre-diagnostic exposures in general population cohorts are generally considered to be etiological rather than prognostic. The observed inverse associations may be partially or wholly driven by selection mechanisms, reverse causation, or residual confounding factors. Consequently, these findings emphasise the importance of conducting rigorous randomised controlled trials to determine causality and define the optimal dosage, intensity and timing of physical activity interventions.

## Figures and Tables

**Table 1:** Characteristics of the studies included in the systematic review.

Reference	Country	Design	Sample Size	Age (Years)	Moment of Measurement	Type of Comparison	Outcomes	
							AM	CM
Bonn SE et al. (2014) [36]	Sweden	Retrospective	4623	63.1 ± 5.1	Post-diagnosis	Categorical	✔	✔
Cadenas-Sánchez C et al. (2025) [37]	Norway	Prospective	404,249	41.6	Pre-diagnosis	Categorical	-	✔
Cannioto RA et al. (2019) [38]	US	Prospective	833	60.6 ± 11.9	Pre- and post-diagnosis	Categorical	✔	✔
Crespo CJ et al. (2008) [39]	Puerto Rico	Prospective	9824	35.0–79.0	Pre-diagnostic	Categorical (Q1–Q4)	-	✔
Di Maso M et al. (2021) [40]	Italy	Retrospective	777	66.0	Pre-diagnosis	Categorical	✔	✔
Elahy V et al. (2025) [41]	US	Prospective	4232	69.0	Pre- and post-diagnosis	Categorical and linear	✔	✔
Friedenreich CM et al. (2016) [42]	Canada	Prospective	830	67.3	Pre- and post-diagnosis	Categorical	✔	✔
Lee DH et al. (2025) [43]	US	Prospective	4779	69.0	Post-diagnosis	categorical	✔	-
Kenfield SA et al. (2011) [44]	US	Prospective	2705	68.3–70.8	Pre- and post-diagnosis	Categorical	✔	✔
Norman A et al. (2002) [45]	Sweden	Prospective	-	>50.0	Pre-diagnosis	Categorical	-	✔
Orsini N et al. (2009) [46]	Sweden	Prospective	-	72.0	Pre-diagnosis	Categorical and linear	-	✔
Rees-Puina E et al. (2025) [47]	US	Retrospective	24,005	67.0 ± 10.0	Post-diagnosis	Categorical	✔	-
Wang Y et al. (2017) [48]	US	Prospective	Predia.: 7328 Postdia.: 5319	71.0	Pre- and post-diagnosis	Categorical	✔	✔
Zhou W et al. (2023) [49]	UK	Prospective	1105	67.9	Pre-diagnostic	Categorical	✔	-
Dai JY et al. (2019) [50]	US	Retrospective	1354	60.0	Pre-diagnosis	Categorical	✔	-

Abbreviations: AM—All-cause mortality in the population with prostate cancer; CM—Prostate cancer-specific mortality; US—United States.

**Table 2:** Results of the systematic review.

Reference	Comparator	Main Comparator	Moment of Measurement	ACM (95% CI)	PCSM (95% CI)	Overall ACM	Overall PCSM
Bonn SE et al. (2014) [36]	<5 vs. ≥5 MET-h/d	Active vs. inactive	Post-diagnosis	HR = 0.63 (95% CI: 0.52–0.77)	HR = 0.78 (95% CI: 0.55–1.11).	↓	=
Bonn SE et al. (2014) [36]	<20 vs. ≥20 min/d walking	Active vs. inactive	Post-diagnosis	HR = 0.70 (95% CI: 0.57–0.86)	HR = 0.61 (95% CI: 0.43–0.87)	↓	↓
Bonn SE et al. (2014) [36]	<1 vs. ≥1 h/d household chores	Active vs. inactive	Post-diagnosis	HR = 0.71 (95% CI: 0.59–0.86)	HR = 0.86 (95% CI: 0.61–1.20)	↓	=
Bonn SE et al. (2014) [36]	<1 vs. ≥1 h/week exercise	Active vs. inactive	Post-diagnosis	HR = 0.74 (95% CI: 0.61–0.90)	HR = 0.68 (95% CI: 0.48–0.94)	↓	↓
Cadenas-Sánchez C et al. (2025) [37]	Walking (work that involves walking, e.g., light industry, inspection)	Sedentary (sedentary work, e.g., office work or assembly of small parts)	Pre-diagnosis	-	Walking: sHR = 0.96 (95% CI: 0.85–1.07)	-	=
Cadenas-Sánchez C et al. (2025) [37]	Walking-and-lifting (work that involves walking and carrying, e.g., mail carrier, construction)	Sedentary (sedentary work, e.g., office work or assembly of small parts)	Pre-diagnosis	-	sHR = 0.92 (95% CI: 0.80–1.05).	-	=
Cadenas-Sánchez C et al. (2025) [37]	Heavy labour (heavy manual labour, e.g., digging or shovelling)	Sedentary (sedentary work, e.g., office work or assembly of small parts)	Pre-diagnosis	-	sHR = 0.94 (95% CI: 0.82–1.09)	-	=
Cannioto RA et al. (2019) [38]	Habitually active (active both before and after diagnosis)	Habitually inactive (inactive both before and after diagnosis)	Pre-diagnosis and Post-diagnosis	HR = 0.48 (95% CI: 0.26–0.87)	HR = 0.52 (95% CI: 0.25–1.10)	↓	=
Crespo CJ et al. (2008) [39]	Vigorous physical activity (≥1 h/day)	Active vs inactive	Pre-diagnosis	-	OR = 0.96 (95% CI: 0.68–1.34)	-	=
Crespo CJ et al. (2008) [39]	Quartile 2 vs. Quartile 1	Active vs inactive	Pre-diagnosis	-	OR = 0.99 (95% CI: 0.64–1.55).	-	=
Crespo CJ et al. (2008) [39]	Quartile 3 vs. Quartile 1	Active vs inactive	Pre-diagnosis	-	OR = 1.34 (95% CI: 0.88–2.05).	-	=
Crespo CJ et al. (2008) [39]	Quartile 4 vs. Quartile 1	Active vs inactive	Pre-diagnosis	-	OR = 1.19 (95% CI: 0.75–1.90)	-	=
Di Maso M et al. (2021) [40]	High physical activity	Low/Moderate physical activity	Pre-diagnosis	HR = 0.79 (95% CI: 0.59–1.07)	HR = 0.95 (95% CI: 0.57–1.59)	=	=
Elahy V et al. (2025) [41]	>15 MET-h/week (Score 2)	<7.5 MET-h/week (Score 0)	Post-diagnosis	HR = 0.80 (95% CI: 0.74–0.87).	HR = 0.67 (95% CI: 0.54–0.84).	↓	↓
Elahy V et al. (2025) [41]	Linear: For every 1-point increase on the physical activity component scale	-	Post-diagnosis	HR = 0.90 (95% CI: 0.86–0.94)	HR = 0.82 (95% CI: 0.73–0.92)	↓	↓
Friedenreich CM et al. (2016) [42]	Quartile 4 vs Quartile 1	Active vs inactive	Pre-diagnosis	HR = 0.58 (95% CI: 0.42–0.79)	HR = 0.65 (95% CI: 0.37–1.13)	↓	=
Lee DH et al. (2025) [43]	3–<9 MET-h/week	<3 MET-h/week	Post-diagnosis	HR = 0.88; 95% CI: 0.74–1.06	-	=	-
Lee DH et al. (2025) [43]	9–<24 MET-h/week	<3 MET-h/week	Post-diagnosis	HR = 0.80 (95% CI: 0.68–0.95)	-	↓	-
Lee DH et al. (2025) [43]	24–<48 MET-h/week	<3 MET-h/week	Post-diagnosis	HR = 0.63 (95% CI: 0.53–0.75)	-	↓	-
Lee DH et al. (2025) [43]	≥48 MET-h/week	<3 MET-h/week	Post-diagnosis	HR = 0.61 (95% CI: 0.51–0.73)	-	↓	-
Kenfield SA et al. (2011) [44]	≥48 MET-h/week	<3 MET-h/week	Post-diagnosis	HR = 0.40 (95% CI: 0.29–0.54)	HR = 0.46 (95% CI: 0.23–0.92)	↓	↓
Kenfield SA et al. (2011) [44]	≥3 h/week	<1 h/week	Post-diagnosis	HR = 0.50 (95% CI: 0.36–0.70)	HR = 0.39 (95% CI: 0.18–0.84)	↓	↓
Kenfield SA et al. (2011) [44]	≥90 min walk	<90 min walk	Post-diagnosis	HR = 0.54 (95% CI: 0.41–0.71)	-	↓	-
Norman A et al. (2002) [45]	High activity	Sedentary	Pre-diagnosis	-	RR = 0.99 (95% CI: 0.89. 1.09)	-	=
Orsini N et al. (2009) [46]	Quartile 4 vs. Quartile 1	-	Pre-diagnosis	-	RR = 0.98 (95% CI: 0.53–1.83).	-	=
Orsini N et al. (2009) [46]	>60 min walking/cycling	20–40 min walking/cycling	Pre-diagnosis	-	RR = 0.72 (95% CI: 0.44–1.18).	-	=
Rees-Puina E et al. (2025) [47]	7.5 a <15.0 MET-h/week	Inactive (0 MET-h/week)	Post-diagnosis	HR = 0.60 (95% CI: 0.49–0.74)	-	↓	-
Wang Y et al. (2017) [48]	≥17.5 MET-h/week	3.5–<8.75 MET-h/week	Pre-diagnosis	HR = 0.88 (95% CI: 0.80–0.97)	HR = 0.95 (95% CI: 0.75–1.21) (overall) HR = 0.63 (95% CI: 0.43–0.91) (low-risk tumours)	↓	↓
Wang Y et al. (2017) [48]	≥17.5 MET-h/week	3.5–<8.75 MET-h/week	Post-diagnosis	HR 0.86 (95% CI: 0.75–0.98)	HR 0.69 (95% CI: 0.49–0.95)	↓	↓
Zhou W et al. (2023) [49]	1.0–1.5 h/day	<1.0 h/day	Pre-diagnosis	HR 0.90; 95% CI: 0.69–1.16	-	=	-
Zhou W et al. (2023) [49]	1.5–2.0 h/day	<1.0 h/day	Pre-diagnosis	HR 0.68 (95% CI: 0.52–0.91).	-	↓	-
Zhou W et al. (2023) [49]	≥2.0 h/day	<1.0 h/day	Pre-diagnosis	HR 0.57 (95% CI: 0.42–0.78).	-	↓	-
Dai JY et al. (2019) [50]	Vigorous physical activity ≥ 1 time per week (combined analysis). Also broken down into 1–3 times/week and > 3 times/week	Vigorous physical activity <1 time per week	Pre-diagnosis	HR = 0.63 (95% CI: 0.42–0.95)	-	↓	-
Dai JY et al. (2019) [50]	Vigorous physical activity 1–3 times/week	Vigorous physical activity <1 time per week	Pre-diagnosis	HR = 0.56 (*p* = 0.008)	-	↓	-
Dai JY et al. (2019) [50]	Vigorous physical activity >3 times/week	Vigorous physical activity <1 time per week	Pre-diagnosis	HR = 0.64 (*p* = 0.09)	-	=	-

Abbreviations: ↓—Inverse association; =—Without statistical significance; 95% CI—95% confidence interval; ACM—All-cause mortality; HR—Hazard ratio; MET—Metabolic equivalent; OR—Odds ratio; PCSM—Prostate cancer-specific mortality; RR—Relative risk; sHR—Subdistribution hazard ratio.

## Data Availability

Data are available upon reasonable request to the corresponding author.

## Supplementary Materials

The following supporting information can be downloaded at https://www.mdpi.com/article/10.3390/healthcare14131998/s1. Table S1. Excluded studies for justified reasons; Table S2. Supplementary characteristics of the studies included in the systematic review; Table S3. Quality assessment of cohort studies; Table S4. Quality of evidence assessment; Figure S1. Albatross plot of selected estimates by outcome and timing of exposure assessment; Section S1. Search strategy.

## Abbreviations

The following abbreviations are used in this manuscript:

AR	Androgen Receptor
GRADE	Grading of Recommendations, Assessment, Development and Evaluation
HR	Hazard Ratio
LTPAQ	Physical Activity Lifetime Questionnaire
OR	Odds Ratio
PC	Prostate Cancer
PRISMA	Preferred Reporting Items for Systematic Reviews and Meta-Analyses
RR	Risk Ratio
