# Supplementary material for: Association Between Physical Activity and Mortality in Men with or at Risk of Prostate Cancer: A Systematic Review
Source: Healthcare (Basel). 2026 Jul 5;14(13):1998. doi: 10.3390/healthcare14131998 (PMC13361563; doi:10.3390/healthcare14131998)
Supplement: Supplementary file 1 [file healthcare-14-01998-s001.zip › healthcare-4406427-supplementary.pdf]

Table S1. Excluded studies for justified reasons.

Reference	Reason
Calle EE et al (2003) [1]	Not exposure of interest
Özdemir K et al (2016) [2]	Not exposure of interest
Lavery JA et al (2024) [3]	Not exposure of interest
Rangul V et al (2018) [4]	Not outcome of interest
Rees-Punia E et al (2024) [5]	Not exposure of interest
Kruk J et al (2025) [6]	Not design of interest

Supplementary references

1. Calle, E.E.; Rodriguez, C.; Walker-Thurmond, K.; Thun, M.J. □verweight, □besity, and Mortality from Cancer in a Prospectively Studied Cohort of US Adults. *New England Journal of Medicine* **2003**, *348*, 1625–1638, doi:10.1056/NEJMoa021423.

2. Özdemir, K.; Keser, I.; Sen, I.; Tan, M.Ö. The Importance of Preventive Physiotherapy in Patients Diagnosed with Prostate Cancer. *JOURNAL OF Urological surgery* **2016**, *3*, 123–126, doi:10.4274/jus.961.

3. Rangul, V.; Sund, E.R.; Mork, P.J.; Roe, □.D.; Bauman, A. The Associations of Sitting Time and Physical Activity on Total and Site-Specific Cancer Incidence: Results from the HUNT Study, Norway. *PLoS One* **2018**, *13*, doi:10.1371/journal.pone.0206015.

4. Lavery, J.A.; Boutros, P.C.; Knight, D.; Tammela, T.; Moskowitz, C.S.; Jones, L.W. Association of Exercise with Pan-Cancer Incidence and □verall Survival. *Cancer Cell* **2024**, *42*, 169–171, doi:10.1016/j.ccell.2023.12.007.

5. Rees-Punia, E.; Masters, M.; Teras, L.R.; Leach, C.R.; Williams, G.R.; Newton, C.C.; Diver, W.R.; Patel, A. V; Parsons, H.M. Long-Term Multimorbidity Trajectories in □lder Adults: The Role of Cancer, Demographics, and Health Behaviors. *Cancer* **2024**, *130*, 312–321, doi:10.1002/cncr.35047.

6. Kruk, J.; Aboul-Enein, B.H.; Gołębiewska, M.E.; Duchnik, E.; Czerniak, U.; Marchlewicz, M. Physical Activity and Cancer Incidence and Mortality: Current Evidence and Biological Mechanisms. *Cancers (Basel).* **2025**, *17*, doi:10.3390/cancers17091410.

Table S2. Supplementary characteristics of the studies included in the systematic review.

Reference	Physical activity assessment method	Sample size by stage	Study duration	Type of cohorts
Bonn SE et al (2014)	Validated physical activity questionnaire	T1a-200, T1b-228, T1c-2262 and T2-1775	Study duration 10-15 years. Analysis time 4.7 years.	Survivor/PC Specific Cohort
Cadenas-Sánchez C et al (2025)	Saltin-Grimby Physical Activity Level Scale (SGPALS)	-	27 years	General Cohort
Cannioto RA et al (2019)	Detailed self-administered epidemiological questionnaire	Stage I-1723, II-1393, III-1024 and unknown-688.	52.7 months	Survivor/PC Specific Cohort
Crespo CJ et al (2008)	Framingham Physical Activity Index	-	Approximately 37 years	General Cohort
Dai JY et al (2019)	Interviews with questionnaire to collect data on frequency of vigorous physical activity	Low risk-544, Intermediate risk-326 and High risk-483	11.3 years	Survivor/PC Specific Cohort
Di Maso M et al (2021)	Self-reported structured questionnaire on work intensity and weekly leisure hours	By Gleason score: Severity (2-6) 395, (7) 164, (8-10) 120 and unknown 98	10 years	Survivor/PC Specific Cohort
Elahy V et al (2025)	Self-report questionnaire (CPS-II cohort)	T1-T2 3.845 and T3-T4 387	14.1 years	Survivor/PC Specific Cohort
Friedenreich CM et al (2016)	Total Lifetime Physical Activity Questionnaire (LTPAQ)	Stage II-642, III- 57, III/IV-76, IV-55	17 years	Survivor/PC Specific Cohort
Lee DH et al (2025)	Validated self-reported AF questionnaires (HPFS)	T1 64%, T2 34% and T3a 2%	15 years	Survivor/PC Specific Cohort
Kenfield SA et al (2011)	Self-administered and validated physical activity questionnaire	T1 50%, T2 36%, T3/T4 2% and missing data 12%	9.7 years for survivors and 7.8 years for deceased	Survivor/PC Specific Cohort
Norman A et al (2002)	Classification of national census work according to physical activity levels by a panel of experts	-	19 years	General Cohort
□rsini N et al (2009)	Self-administered and validated physical activity questionnaire	-	9-10 years	General Cohort
Rees-Puina E et al (2025)	Harmonised self-report questionnaires that measure moderate-to-vigorous physical activity	In situ 9-12%, localized 42-48%, distant 10-14% and unknown 4-9%	10,9 years	Survivor/PC Specific Cohort
Wang Y et al (2017)	Self-administered biennial follow-up questionnaires	Low risk (Gleason 2-7 and T1-T2) 3,463; High risk (Gleason 8-10, T3-T4 or N1) 1,350	19-20 years	Survivor/PC Specific Cohort

Zhou W et al (2023)	Triaxial wrist accelerometer (Axivity AX3)	-	7.4 years	Survivor/PC Specific Cohort

Table S3. Quality assessment of cohort studies.

Reference	1	2	3	4	5	6	7	8	9	10	11	12	13	14	Overall
Bonn SE et al (2014)	Yes	Yes	Yes	Yes	No	Yes	Yes	Yes	Yes	No	Yes	No	Yes	Yes	Poor
Cadenas-Sánchez C et al (2025)	Yes	Yes	Yes	Yes	No	Yes	Yes	Yes	Yes	No	Yes	No	Yes	Yes	Poor
Cannioto RA et al (2019)	Yes	Yes	Yes	Yes	No	Yes	Yes	Yes	Yes	No	Yes	No	Yes	Yes	Poor
Crespo CJ et al (2008)	Yes	Yes	Yes	Yes	No	Yes	Yes	Yes	Yes	No	Yes	No	Yes	Yes	Poor
Dai JY et al (2019)	Yes	Yes	Yes	Yes	No	Yes	Yes	Yes	Yes	No	Yes	No	Yes	Yes	Poor
Di Maso M et al (2021)	Yes	Yes	Yes	Yes	Yes	Yes	Yes	Yes	Yes	No	Yes	No	Yes	Yes	Fair
Elahy V et al (2025)	Yes	Yes	Yes	Yes	No	Yes	Yes	Yes	Yes	No	Yes	No	Yes	Yes	Poor
Friedenreich CM et al (2016)	Yes	Yes	Yes	Yes	No	Yes	Yes	Yes	Yes	Yes	Yes	Yes	Yes	Yes	Good
Lee DH et al (2025)	Yes	Yes	Yes	Yes	No	Yes	Yes	Yes	Yes	Yes	Yes	No	Yes	Yes	Fair
Kenfield SA et al (2011)	Yes	Yes	Yes	Yes	No	Yes	Yes	Yes	Yes	Yes	Yes	No	Yes	Yes	Fair
Norman A et al (2002)	Yes	Yes	Yes	Yes	No	Yes	Yes	Yes	Yes	Yes	Yes	No	Yes	No	Poor
□rsini N et al (2009)	Yes	Yes	No	Yes	No	Yes	Yes	Yes	Yes	No	Yes	No	Yes	Yes	Poor
Rees-Puina E et al (2025)	Yes	Yes	Yes	Yes	No	Yes	Yes	Yes	Yes	No	Yes	No	Yes	Yes	Poor
Wang Y et al (2017)	Yes	Yes	Yes	Yes	No	Yes	Yes	Yes	Yes	No	Yes	No	Yes	Yes	Poor
Zhou W et al (2023)	Yes	Yes	No	Yes	No	Yes	Yes	Yes	Yes	No	Yes	No	Yes	Yes	Poor

Assessment of risk of bias for observational studies with 'Study Quality Assessment Tools'. Green: yes (Y); Red: no (N); Yellow: some concerns, including cannot determine, not reported; not applicable.

Items

1. Is the research question or objective clearly stated in this document?
2. Was the study population clearly specified and defined?
3. Was the participation rate of eligible subjects at least 50%?
4. Were all subjects selected or recruited from the same or similar populations (and within the same time period)? Were the inclusion and exclusion criteria for participation in the study pre-specified?
5. Was the sample size justified, and was a description of the power or estimates of variance and effect provided?
6. For the analyses in this document, were the exposures of interest measured before the outcomes?
7. Was the timeframe long enough to expect a reasonable association between the exposure and the outcome, if one existed?
8. For exposures that may vary in quantity or level, did the study examine different levels of exposure in relation to the outcome (e.g. exposure categories or exposure measured as a continuous variable)?
9. Were the exposure measures (independent variables) clearly defined, valid, reliable and consistently applied to all study participants?
10. Were the exposure(s) assessed more than once over time?
11. Were the outcome measures (dependent variables) clearly defined, valid, reliable and consistently applied to all study participants?
12. Were the outcome assessors blinded to the exposure status of the participants?
13. Was the loss to follow-up after baseline 20% or less?
14. Was major potential confounding variables measured and statistically adjusted for their impact on the relationship between the exposure(s) and the outcomes?

Table S4. Quality of evidence assessment.

Certainty assessment							Certainty
**№ of studies**	**Study design**	**Risk of bias**	**Inconsistency**	**Indirectness**	**Imprecision**	**Other considerations**	
**A. All-cause mortality in prostate cancer**							
**11**	Observational	Serious	Not serious	Not serious	Serious	Dose-response gradient	Very Low
**B. Prostate cancer-specific mortality**							
**7**	Observational	Serious	Serious	Not serious	Serious	None	Very Low

Figure S1. Albatross plot of selected estimates by outcome and timing of exposure assessment.

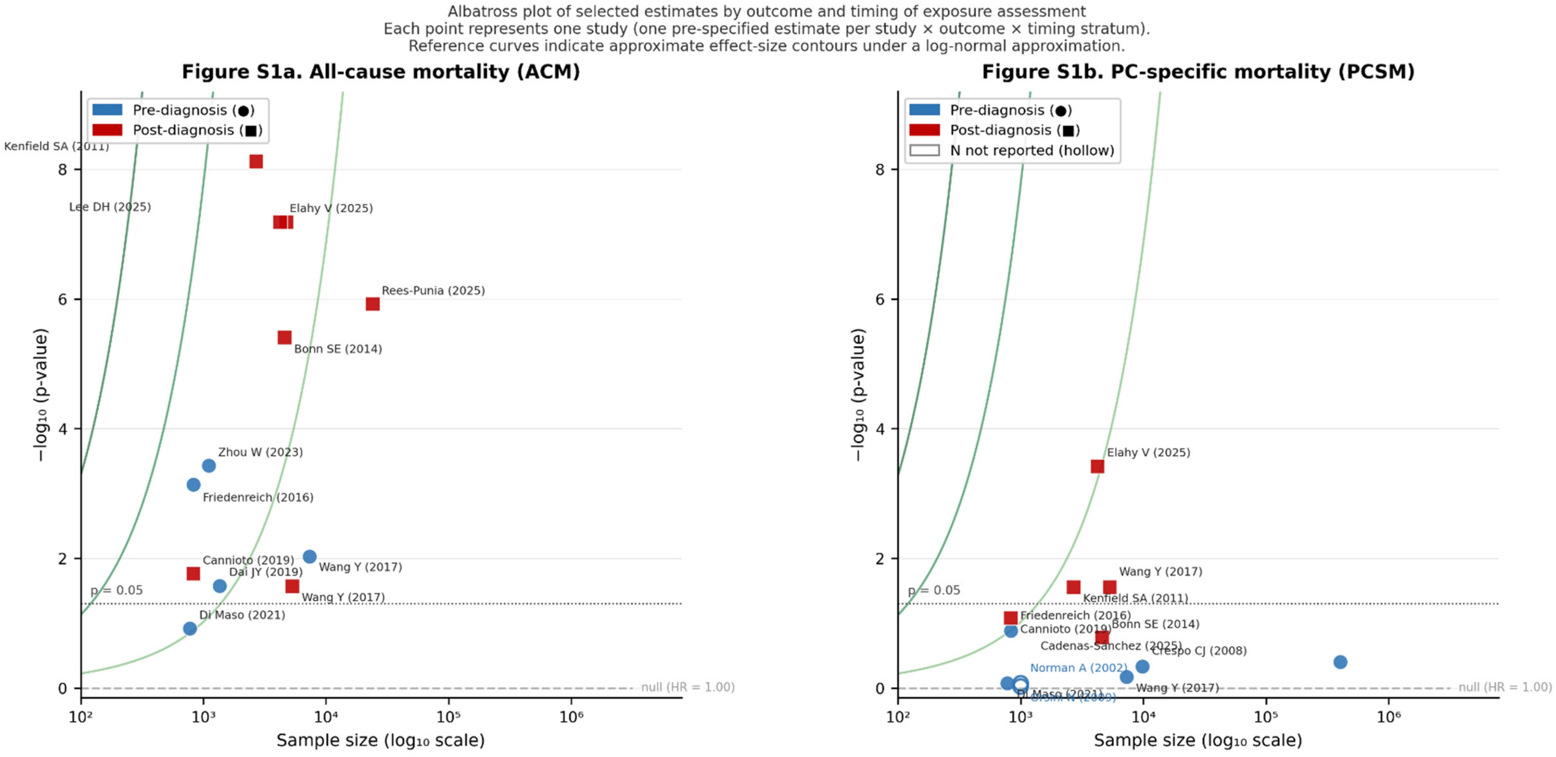

Section S1. Search strategy.

- Medline (via PubMed)

("prostate cancer"[All Fields] □R (("prostat"[All Fields] □R "prostate"[MeSH Terms] □R "prostate"[All Fields] □R "prostates"[All Fields] □R "prostatic"[All Fields] □R "prostatism"[MeSH Terms] □R "prostatism"[All Fields] □R "prostatitis"[MeSH Terms] □R "prostatitis"[All Fields]) AND ("cancer s"[All Fields] □R "cancerated"[All Fields] □R "canceration"[All Fields] □R "cancerization"[All Fields] □R "cancerized"[All Fields] □R "cancerous"[All Fields] □R "neoplasms"[MeSH Terms] □R "neoplasms"[All Fields] □R "cancer"[All Fields] □R "cancers"[All Fields]))) AND ("physical activity"[All Fields] □R "leisure-time physical activity"[All Fields] □R "occupational activity"[All Fields] □R "active behavio*"[All Fields] □R "sedentary behavio*"[All Fields]) AND ("mortality"[MeSH Terms] □R "mortality"[All Fields] □R "mortalities"[All Fields] □R "mortality"[MeSH Subheading] □R "all-cause mortality"[All Fields] □R ("mortality"[MeSH Subheading] □R "mortality"[All Fields] □R "survival"[All Fields] □R "survival"[MeSH Terms] □R "survivability"[All Fields] □R "survivable"[All Fields] □R "survivals"[All Fields] □R "survive"[All Fields] □R "survived"[All Fields] □R "survives"[All Fields] □R "surviving"[All Fields]) □R ("death"[MeSH Terms] □R "death"[All Fields] □R "deaths"[All Fields])) AND ("hepatoma res"[Journal] □R "hr"[All Fields] □R "hazard ratio"[All Fields] □R "risk ratio"[All Fields] □R "odds ratio"[All Fields] □R ("epidemiology"[MeSH Subheading] □R "epidemiology"[All Fields] □R "incidence"[All Fields] □R "incidence"[MeSH Terms] □R "incidences"[All Fields] □R "incident"[All Fields] □R "incidents"[All Fields]) □R "relative risk"[All Fields])

- Scopus

TITLE-ABS-KEY(("prostate cancer" □R (prostate AND cancer)) AND ("physical activity" □R "leisure-time physical activity" □R "occupational activity" □R "active behavio*" □R "sedentary behavio*") AND (mortality □R "all-cause mortality" □R survival □R death) AND (HR □R "hazard ratio" □R "risk ratio" □R "odds ratio" □R incidence □R "relative risk"))

- Web of Science

ALL=(("prostate cancer" □R (prostate AND cancer)) AND ("physical activity" □R “leisure-time physical activity” □R “occupational activity” □R “active behavio*” □R “sedentary behavio*”) AND (mortality □R “all-cause mortality” □R survival □R death) AND (HR □R "hazard ratio" □R "risk ratio" □R "odds ratio" □R incidence □R "relative risk"))

- Grey literature

□pen search.
